# A novel approach to monitoring rehabilitation progress in atrophic muscle using contactless measurement of free oscillations and advanced modal analysis

**DOI:** 10.3389/fbioe.2025.1496739

**Published:** 2025-08-01

**Authors:** Agnieszka Tomaszewska, Milena Drozdowska, Piotr Aschenbrenner

**Affiliations:** ^1^ Department of Structural Mechanics, Faculty of Civil and Environmental Engineering, Gdansk University of Technology, Gdansk, Poland; ^2^ Department of Biomechanics and Sports Engineering, Gdansk University of Physical Education and Sport, Gdansk, Poland

**Keywords:** muscle vibrations, muscle atrophy, anterior cruciate ligament, rectus femoris muscle, rehabilitation progress monitoring, eigen system realization algorithm, human health

## Abstract

This study shows a novel approach for monitoring muscle functional recovery through the analysis of its free vibrations. Contactless measurements of free-decay vibrations are carried out, and by applying an advanced experimental modal analysis, the oscillations’ frequencies are identified with high accuracy. A laser displacement sensor is used to capture the oscillations at a selected muscular point. The efficacy of the approach is demonstrated in the case of functional status recovery of the rectus femoris muscle, which became atrophic after anterior cruciate ligament (ACL) reconstruction. Three ACL subjects are examined, and the muscle is analyzed in two states: voluntary tension and relaxation. The analysis shows significant changes in the natural frequency of the rectus femoris throughout the muscle recovery process, with the frequency gradually approaching that of the same muscle in the subject’s contralateral (reference) leg. Moreover, the relative difference in natural frequencies identified between the ACL-affected leg and the reference leg tends, over time and within rehabilitation, to align with the values observed in healthy reference subjects. This approach demonstrates the potential to reliably measure the natural frequency of a muscle. The contactless nature of the measurement ensures that vibrations remain unaffected by any external probes. This approach shows practical potential for monitoring muscular rehabilitation progress, assessing the muscular functional status of muscles in patients with dysfunction, and evaluating muscular readiness for participation in sports competitions.

## 1 Introduction

Vibration-based structural health monitoring (VBSHM) was originally developed in mechanical and civil engineering (Fritzen, 2005). A major group of methodologies relies on modal characteristics analysis, which in linear systems are controlled only by system parameters such as mass, stiffness, and tension state (e.g., pre-stress). Thus, modal parameters can serve as indicators of system evolution. The non-invasiveness of the VBSHM makes the techniques particularly well-suited for biomechanics, providing safe and quick *in vivo* measurements. In recent years, there has been a growing interest in the use of VBSHM techniques in biomechanics, both in the context of clinical diagnostics and for monitoring the progress of rehabilitation or training adaptations. Vibration methods are used, among others, to assess muscle and tendon stiffness, detect structural changes after injuries, and also for the early detection of overloads and dysfunctions in the musculoskeletal system (Torres et al., 2023). Studies show that dynamic characteristics of tissues (such as resonance frequency or vibration damping) correlate with physiological parameters such as muscle tension, fatigue, or elasticity (Zhang et al., 2021). These methods have found application in the study of biological tissues, including muscles, tendons, and the musculoskeletal system (Zhang et al., 2021; Vien et al., 2022). For example, vibration-based analysis of stiffness and tension of the Achilles tendon is considered by Salman and Sabra (2015) and Martin et al. (2018), and that of other soft tissues is considered by Silver et al. (2021). In the latter paper, the authors declare that vibrational optical coherence tomography can be used to detect a decrease in tissue tension modulus by observing a change in the tissue’s resonant frequency. Additionally, full-field measurement of vibrations using electronic speckle pattern interferometry is used to study the relation between the facial vibrations and singing (Frič et al., 2021) or identify constitutive models of soft tissues covering the forearm (Atashipour and Baqersad, 2023).

In this study, VBSHM is employed as a non-invasive methodology for monitoring the rehabilitation progress of atrophic muscle. A common phenomenon specifically considered is the atrophy of the rectus femoris (RF) muscle following anterior cruciate ligament (ACL) reconstruction (ACLR) of the knee joint. Various sources denote 30 to 78 ACL ruptures per 100,000 person-years (Gans et al., 2018). Moreover, restoring the pre-injury strength of the quadriceps is challenging, even with intensive rehabilitation (Mattacola et al., 2002; Ithurburn et al., 2018). Therefore, there is a need for a reliable, non-invasive, and preferably straightforward method to monitor muscle recovery. Currently available methods for assessing muscular functional status include evaluating the strength of muscle groups acting on the knee joint through torque measurement (Mattacola et al., 2002); assessing muscle tone or local stiffness using myotonometry (Aird et al., 2012; Stefaniak et al., 2022); measuring muscle elasticity with shear wave elastography (SWE) (Nowicki and Dobruch-Sobczak, 2016; Wu et al., 2020); and identifying neuromuscular characteristics using functional electrical stimulation (FES) (Suzuki et al., 2024; Matsui et al., 2014). However, these methods have limitations. In the joint torque measurement, it is challenging to isolate a single muscle, and furthermore, the test cannot be conducted shortly after an injury or operation. Myotonometry measures muscle vibrations in direct contact with the tissue, which impacts the measurement results due to the gentle nature of soft-tissue vibrations. Additionally, the SWE equipment is expensive and requires an expert operator, and electrical stimulation of a muscle may be uncomfortable for the patient.

The article presents an innovative approach for monitoring the rehabilitation progress of the RF muscle, which becomes atrophic after ACLR, using contactless measurement of the muscle’s free vibrations combined with advanced modal analysis. The vibrations are induced by a tap on the muscle, right above the measurement point, and the muscle response is captured by a laser displacement sensor. The muscle’s free vibration frequency, or natural frequency, following vibration theory nomenclature, is identified using the advanced experimental modal analysis technique known as the eigensystem realization algorithm (ERA) (Juang and Pappa, 1985). Pre-injury symmetry of the RF muscles in both legs of a patient is assumed, and thus, muscle rehabilitation progress is tracked by comparing the oscillation frequencies between the repaired ACL leg and the healthy reference leg. The efficacy of this methodology is demonstrated through a case study involving three subjects who were analyzed at several time points spaced a few weeks apart. The Biodex System 4 PRO device was simultaneously used to capture the torque of the knee extensor muscles as a reference, a well-established method used for the muscle group’s strength assessment. Part of the research has been presented in a preprint (Tomaszewska et al., 2023).

## 2 Methods

### 2.1 Goal and research hypothesis

The goal of the paper is to propose a novel indicator of muscle rehabilitation progress based on the muscle’s modal characteristic—the natural frequency. The concept is initially verified on three subjects who experienced ACLR (ACL subjects) and RF muscle atrophy related to the operation. The rehabilitation progress indicator is defined by a relative difference between the natural frequencies of the RF muscle in the reference and the ACL legs of a subject, using the following Formula 1:

$$D_{f}=\frac{\left(f_{r}-f_{a}\;\right)}{\;f_{r}},$$ (1)

where *f*_*r*_ and *f*_*a*_ denote the mean values of the natural frequencies of the RF muscle in the reference and ACL legs in the same subject, respectively. In addition, for healthy subjects, the absolute value of *D*_*f*_ is considered to obtain reference data.

Research hypothesis: the *D*_*f*_ value in ACL subjects is the highest after operation, and within the rehabilitation process, it tends to the mean value of *D*_*f*_ determined for the reference group.

### 2.2 Identification of the muscle’s natural frequency

A highly accurate modal identification method is needed for detecting changes in the muscle’s natural frequency as these changes are expected to be relatively small. Although the Fourier transform is sometimes used to identify natural frequencies, it provides only general information on the frequencies present in the analyzed signal (related to the system itself but also to input and noise). Advanced modal identification techniques exclusively select the characteristics of the considered system. The ERA technique is suitable for short-duration, fast-decaying vibration signals, such as those generated in the muscle tissue by a single mechanical impulse. Its effectiveness in identifying dynamic characteristics of systems, such as natural frequencies, mode shapes, and damping ratios, is discussed by Tomaszewska and Szafrański (2020) and Tomaszewska et al. (2020). High identification accuracy is achieved using a stabilization diagram based on different orders of the analysis, effectively eliminating the bias error.

The ERA is formulated on matrix operations. First, the state-space representation of a linear dynamic system should be introduced. For a discrete-time system, it is reflected using a system of Equation 2:

$$\left\{\begin{array}{l} \dot{x}\left(t\right)=A_{c}x\left(t\right)+B_{c}u\left(t\right)\\ y\left(t\right)=C_{c}x\left(t\right)+D_{c}u\left(t\right) \end{array}\right.,$$ (2)

where **A**, **B**, **C**, and **D** are the state, input, output, and transmission matrices, respectively, and **u**(t), **x**(t), and **y**(t) are the input, state, and output vectors, respectively. The searched natural frequencies of a system are included in the **A** matrix. This matrix is constructed following a dedicated procedure, which starts with a Hankel matrix definition using the measured vibration signal. Parts of a time-domain decaying signal (triggered by an impulsive input) form a Hankel matrix of Markov parameters $y_{k}$ , see Formula 3. Considering a single output **y**(t), the matrix includes subsequent signal samples at discrete time points *k*.

$$H\left(k-1\right)=\left[\begin{array}{cccc} y_{k} & y_{k+1} & \cdots & y_{k+\beta -1}\\ y_{k+1} & y_{k+2} & \cdots & y_{k+\beta }\\ \vdots & \vdots & \ddots & \vdots \\ y_{k+\alpha -1} & y_{k+\alpha } & \cdots & y_{k+\alpha +\beta -2} \end{array}\right]\in R^{\alpha \times \beta },$$ (3)

where α and β are the parameters to determine the size of the Hankel matrix. Next, singular value decomposition (SVD) is performed on the Hankel matrix to obtain the three matrices **S**, **U**, and **V**: the matrix made of singular values of the Hankel matrix, arranged in a non-increasing order, and two non-singular orthonormal matrices, made of vectors corresponding to singular values of the **S** matrix, respectively (see Formula 4).

$$\text{SVD }H\left(0\right)=\text{US}V^{T}.$$ (4)

The crucial part of the ERA procedure is to select the model order *n*. The elements lying in *n* first columns and *n* first rows of the matrix **S** contain significant singular values of the system (1). The reduced matrices $U_{n},S_{n},{\text{and}\;V}_{n}$ are formed accordingly, and they are used to reconstruct the Hankel matrix $\tilde{H}$ , partially filtered out of noise. The state matrix $\hat{A}$ is estimated using the following Equation 5:

$$\hat{A}=S_{n}^{-1/2}U_{n}^{T}\tilde{H}\left(1\right)V_{n}S_{n}^{-1/2},$$ (5)

which allows for the identification of modal parameters by solving the eigenvalue problem of the $\hat{A}$ matrix $\hat{A}\Phi =\Phi \hat{\Lambda }$ . Natural frequencies are finally extracted from the diagonal matrix $\hat{\Lambda }$ . The algorithm returns frequencies involved in the signal, and to identify the system characteristics, the modal assurance criterion (*MAC*) and damping criterion are verified, where the *MAC* is defined as the coherence between two sets of modal vectors, and the damping criterion excludes modes with a non-positive damping ratio or a value higher than 1. It is accepted that *MAC* > 0.95 points at the natural frequency of the system. Typically, a stabilization diagram is plotted to observe whether all the identified frequencies with the *MAC* and damping criterion are satisfied or not. Details of the ERA technique are described by Juang and Pappa (1985).

### 2.3 Subjects

The study includes three subjects after ACLR and four reference subjects. The subjects are all slim and are sports amateurs. Their body composition was measured by bioelectrical impedance analysis (InBody720, Biospace, Korea), and the percentage of fat in the total body mass was calculated. The ACL subjects have the following characteristics:

• Subject 1: female, age 46, BMI 19.0, body fat 18%, and first vibration test 5 months after ACLR.
• Subject 2: female, age 21, BMI 21.8, body fat 21%, and first vibration test 1 month after ACLR.
• Subject 3: male, age 23, BMI 25.8, body fat 5%, and first vibration test 3 weeks after ACLR.

The reference subjects have the following characteristics:

• Subject 4: male, age 24, BMI 23.6, and body fat 12%.
• Subject 5: female, age 23, BMI 19.7, and body fat 25%.
• Subject 6: male, age 23, BMI 20.4, and body fat 18%.
• Subject 7: male, age 24, BMI 21.6, and body fat 17%.

All ACL subjects underwent ACL reconstruction within 3 months post-injury and experienced quadriceps atrophy immediately after reconstruction. They started rehabilitation 1 month after ACLR. The subjects were measured at three or four time points; subjects 1 and 3 were measured at irregular time intervals, while subject 2 was measured at regular 6-week intervals. Detailed timelines are shown in Supplementary Tables S1–S3. All subjects provided informed consent for the research.

### 2.4 Data collecting

The muscular vibrations were measured at a selected point using a laser displacement sensor optoNCDT (Micro-Epsilon, Germany). The red-light ILD1402-600 model of 80-µm resolution was used in the first three time points for subject 1. Subsequently, the sensor model was replaced with a blue-light ILD1750-200BL model, which is safer for biological subjects, offering a 15-µm resolution (purchased from P.P.H. WObit E.J. Ober s.c., Poland). The sensor change did not affect the results, which were checked in a preliminary study with simultaneous measurements taken using the two sensors. The frequency of vibrations (which is the subject of interest in this study) measured using the two sensors was the same. The measurement rate was set to 100 Hz. A laser displacement sensor measures the absolute distance of the observed point from the sensor. In the study, the observed muscular point oscillates around its rest position, which was determined before inducing the vibrations. In the analysis, this rest position was subtracted from the measured oscillation signals, and finally, oscillations around 0 were analyzed in ERA. Gavronski et al. (2007) determined the natural frequency of RF muscles in junior triathletes to be 12.59 ± 0.99 Hz in the relaxed state and 16.37 ± 1.60 Hz while in contraction. Free damped muscular vibrations were analyzed in their study using a myotonometer. Vibrations were induced using a probe and measured by the same probe kept in contact with the muscle. Taking these values as a reference, in the present study, the signals were initially pre-filtered using a fourth-order elliptic band-pass filter with a range of 6–20 Hz, 0.05 dB peak-to-peak passband ripple, and 40 dB stopband attenuation. The data were analyzed using the MATLAB environment.

**FIGURE 1:**
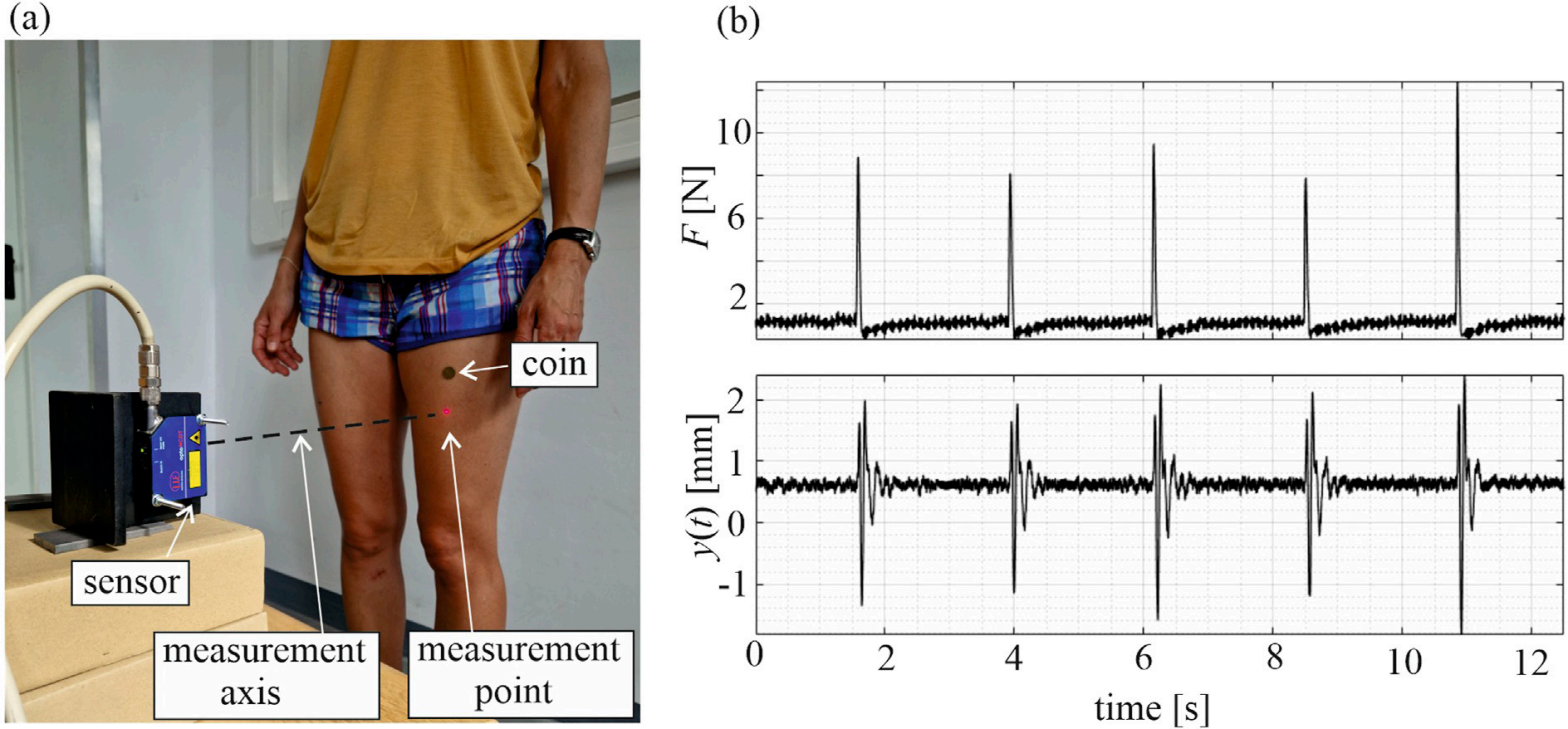
**(a)** View of the experimental stand. **(b)** Signals measured in ACL leg—applied force *F* and vibration response *y* of the rectus femoris muscle in a tense state; subject 1, second time point.

Subjects were positioned in a standing, subjectively symmetrical posture, with their backs stabilized by a chair to minimize body sway. Displacement time series were recorded in the sagittal plane in the horizontal direction. After conducting preliminary measurements and signal analysis at different points of the muscles in both legs, a point in the middle distance between the anterior superior iliac spine and the knee axis was selected for vibration tracking. An overview of the measurement setup is presented in Figure 1a. The muscle vibrations were induced by a tapping impulse made using a modal hammer (model 086D05, PCB Piezotronics) applied 3 cm above the measurement point, allowing for the measurement of the force applied to the muscle. The tapping was applied by a trained operator, who tried to maintain a similar load value in each tap. The signals from the sensor and the hammer were collected using the universal amplifier HBM 1-MX840B (Hottinger Brüel and Kjær) connected to a computer. This enabled real-time observation of the applied forces and the corresponding muscular responses, allowing on-the-fly decision about force repetition—usually necessary when the applied force was too low and the observed muscular response barely noticeable. Fifteen valid impulses and muscular responses were collected for each tested case. To prevent hematoma formation, the tapping point was covered with a small coin, attached to the skin with wig tape. The study examined both muscles (rectus femoris in both legs) in two states: the relaxed state and the tense state (voluntary maximal contraction).

In addition, knee joint torque was measured in both legs as a gold standard for assessing the strength of a group of muscles. Biodex System 4 PRO (Biodex Medical System Inc., Shirley, NY, United States) was used. Isometric unilateral extension test with 90° knee bend was employed, maintaining a 30 s relaxation time and 5 s quadriceps contraction time. Three repetitions were performed each time to collect peak values. For ACL subjects, these measurements started from the second time point because in the first time point, the knee was not ready for such loading.

### 2.5 Statistical analysis

The collected data were subjected to standard statistical analysis. Descriptive statistics, such as mean values and standard deviations of natural frequencies identified for each subject, were calculated. The research hypothesis was tested using the two-way ANOVA test with a significance level of *p* = 0.05. The normality of the *D*_*f*_ distribution among the reference and ACL groups was checked using the Shapiro–Wilk test and the equal variance test (Brown–Forsythe).

## 3 Results

### 3.1 Frequency analysis

**FIGURE 2:**
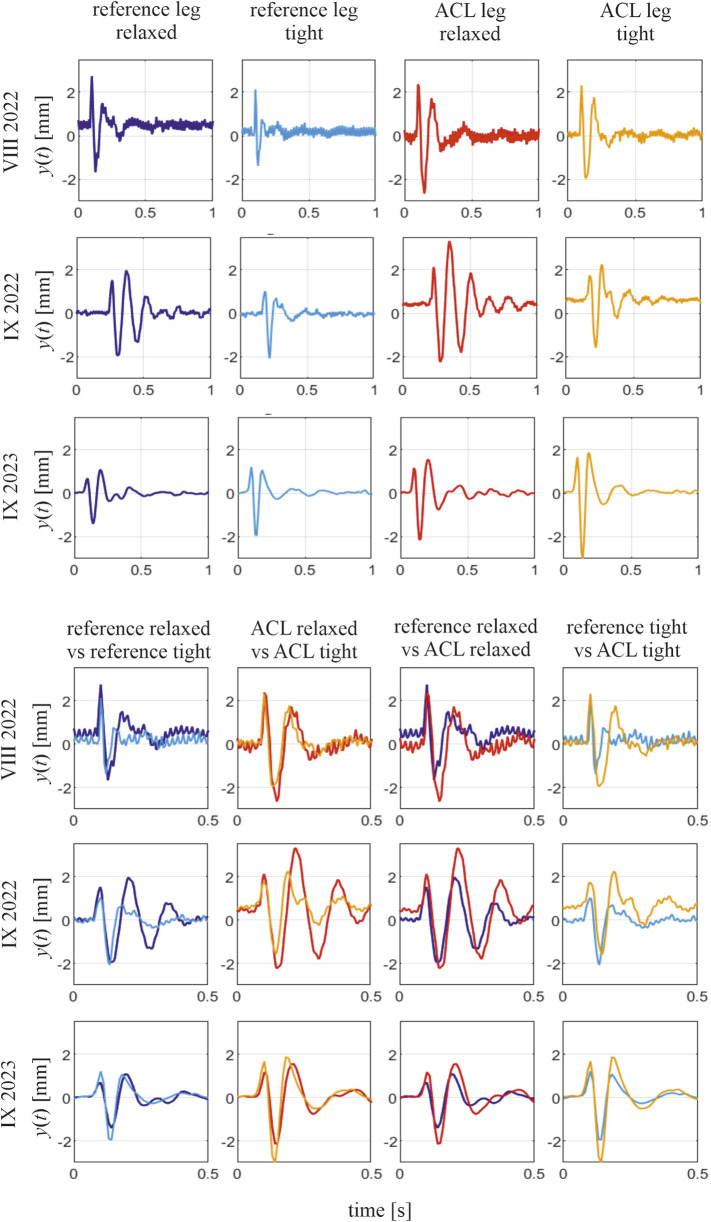
Selected responses of the RF muscle to tapping impulse and comparison of the responses in different muscular states; subject 1, three time points. In the comparisons, the two presented signals have the first magnitude aligned with time to conveniently present different periods of vibrations in them.

Selected signals collected for subject 1 are depicted in Figures 1, 2. Force signals applied to the RF muscle in the ACL leg in a tense state, along with the corresponding muscular responses, are illustrated in Figure 1b, and exemplary muscular vibration response signals at different time points are presented in Figure 2, with comparisons between the tense and relaxed muscular states and additionally between the ACL and reference legs. Notably, some variations in peak force did not cause a significant change in the identified natural frequency of a muscle.

**FIGURE 3:**
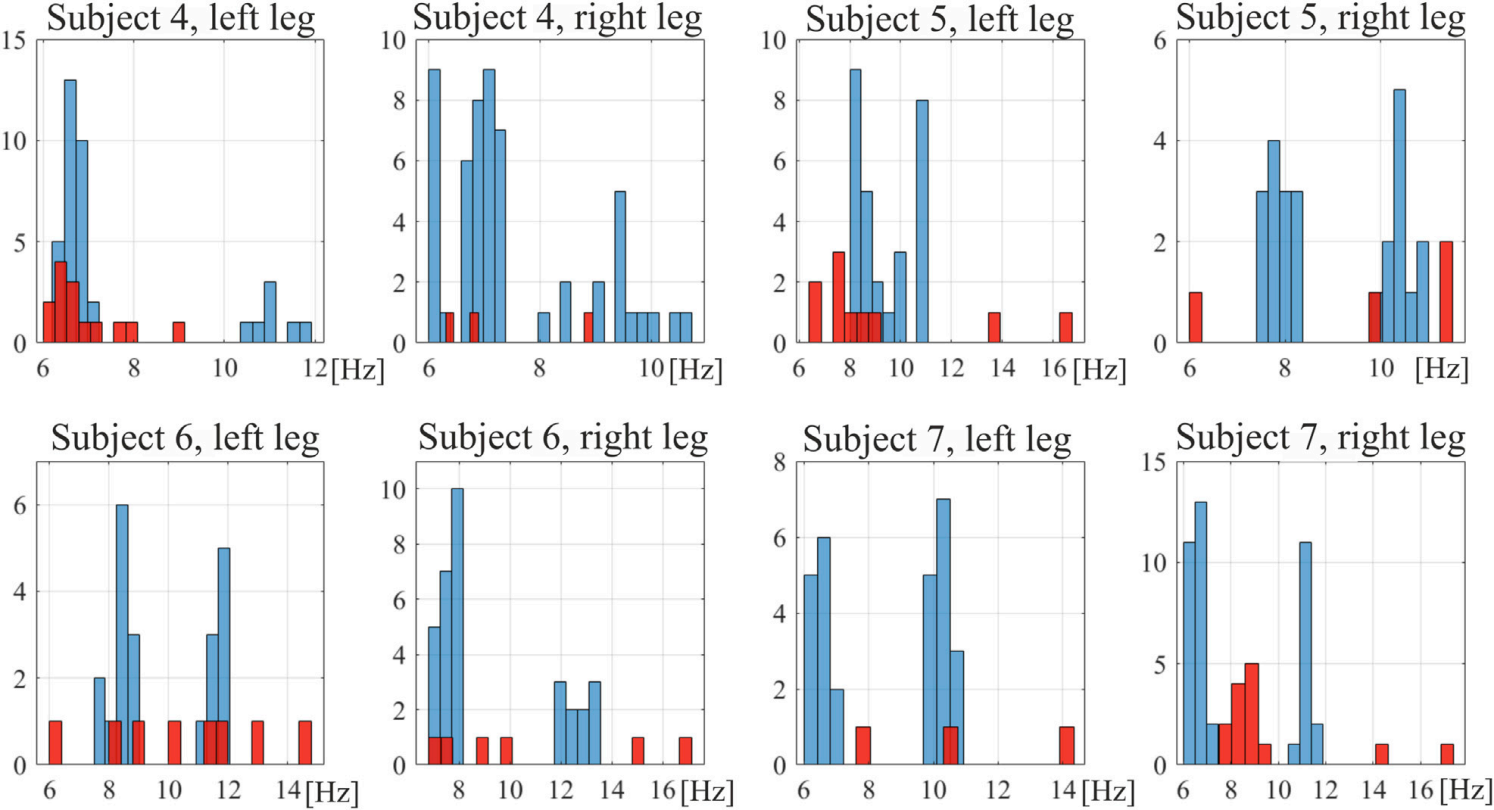
Histograms of natural frequencies of the rectus femoris muscle in reference subjects. Blue bars, relaxed muscle; red bars, tensed muscle.

**FIGURE 4:**
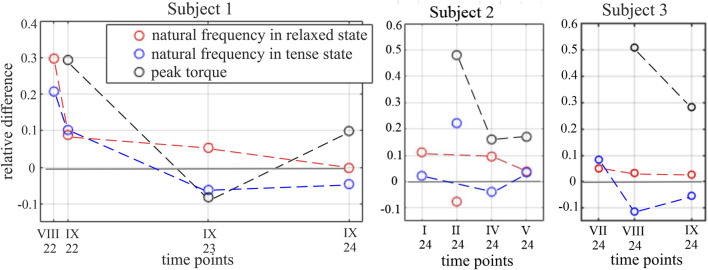
*D_f_* indices and relative difference between peak torque in the knee joint in the reference and ACL legs for subjects 1–3. The trends of the value change are marked by dashed lines. The results for subject 2, II.2024, are excluded from the analysis as outliers.

Each signal of induced vibrations was considered in ERA. Due to multiple force repetitions and a few orders of ERA being considered (2, 4, and 6), a set of results, i.e., the value of natural frequency of a muscle, was obtained for each case. In general, 3–54 results were collected in different sets. The lower number was observed in the tense muscular state as strongly tensed muscles exhibit high damping and very short time responses—in some cases with virtually no detectable vibrations. In such cases, a limited number of signals provided information regarding the natural frequency. All identified frequencies for reference subjects are depicted in histograms in Figure 3. One can observe two distinctive bands in the relaxed muscular state at approximately 8 Hz and 11 Hz, which may suggest two separate modes of the muscles. However, it does not occur for subject 5 (left leg) and is not obvious for subject 4 (right leg). Therefore, to calculate the *D*_*f*_ indicator of rehabilitation progress, all frequencies determined in the considered band (6–20 Hz) are taken to calculate the average frequency of a muscle in a given state. The oscillation frequencies identified for all subjects, in both legs, across all time points, together with peak torque values and *D*_*f*_ values are presented in Supplementary Tables S1–S4. Relative differences between the frequencies obtained for ACL and reference RF muscles in ACL subjects, *D*_*f*_, are depicted in Figure 4. One may notice a more regular trend of *D*_*f*_ decrease for all ACL subjects in the relaxed muscular state; thus, the research hypothesis is verified for this state.

### 3.2 Verification of research hypothesis

The *D*_*f*_ results obtained for each subject at each time point were subjected to two-way ANOVA analysis. While three time points are available for subject 3, the same number of points were also considered for subjects 1 (last three time points are included) and 2. Although the data did not pass the Shapiro–Wilk normality test, they passed the equal variance test (Brown–Forsythe), *p* = 0.863, which allows continuation of the analysis. In the ANOVA test, one factor was the group (reference or ACL), while the second was the time point of ACL subjects’ measurements (P1, P2, and P3). In the ACL group, the following mean values of *D*_*f*_ were obtained for the subsequent time points P1, P2, and P3: *D*_*f*_ (P1) = 0.0830, *D*_*f*_ (P2) = 0.0602, and *D*_*f*_ (P3) = 0.0208. For the reference subjects, the average *D*_*f*_ = 0.0382. The significance of mean differences between measurement time points was *p* = 0.138, and in the case of differences between groups, it was *p* = 0.192. The value of the ANOVA test for the tested hypothesis was *p* = 0.232.

## 4 Discussion

The aim of this paper is to present a novel methodology for monitoring muscle rehabilitation progress. Although a limited number of subjects are presented, the results are promising.

Notable differences are observed between the vibration signals produced by a single muscle in different tension states and at different time points during the rehabilitation process. First, across all time points and all subjects, the short-lasting signals of decaying character, which are responses to the applied tapping, reveal the characteristic difference when measured for the tense and relaxed muscular state. In other words, the signals collected in the tense muscular state reveal shorter periods of these decaying vibrations than those collected in the relaxed state of the same muscle. This is illustrated by the example signals collected for subject 1 at three time points (see Figure 2). This indicates a higher natural frequency of the muscle in the tense state, aligning with the theory of vibrations that between two similar systems, the one with greater stiffness has a higher natural frequency. However, the potential of using a muscular natural frequency as an indicator of muscle functional status becomes apparent when comparing the frequencies of the ACL and reference legs in similar tension states. The periods of the induced free vibrations of the rectus femoris in the ACL and reference legs tend to equalize as rehabilitation progresses. This is visible in all ACL subjects when considering either the tense or relaxed states of both the compared muscles. This also induces a gradual equalization of natural frequencies between the observed muscles over time. This can be noticed in the numerical results presented in Supplementary Material and Figure 4, where the gradual reduction in the difference in natural frequencies between the ACL and reference legs can be easily noticed. The indicator of the functional status improvement of the RF muscle in the ACL leg is represented by the relative difference, *D*_*f*_, between the natural frequency identified for this muscle in the ACL leg, *f*_*a*_, and the frequency for a twin muscle in a reference leg. The indicator is calculated at each specific time point and in each particular muscular state (relaxed or tense). As shown in Figure 4, the value of the *D*_*f*_ indicator for all ACL subjects tends to approach 0 over time, i.e., indicating rehabilitation progress.

An exemplary stabilization diagram of the ERA analysis of vibration signals is depicted in Supplementary Material. The power spectral density (PSD) function is presented there, showing a considerably wide band of oscillation spectrum (see Supplementary Figure S1). In all cases, the muscles exhibited such wide first bands of the oscillation spectrum. This is clearly visible in the results of natural frequencies of the RF muscles of reference subjects (Figure 3). Within this band, several frequencies were identified as the system characteristics. On the other hand, relatively high changeability in natural frequencies was observed across different considered cases for a single subject. This, combined with signal noise and potential system nonlinearity, led to the decision to consider the average values of frequencies identified in a band of 6 Hz–20 Hz. These results are presented in tables in the Supplementary Material. The diversity of results is indicated by relatively high values of standard deviations. Nonetheless, the natural frequencies presented for the reference leg are consistent with those previously reported for junior triathletes by Gavronski et al. (2007), which were measured using a myotonometer.

Differences in natural frequencies between the two legs are noticeable in both of the considered muscular tension states. However, the proposed indicator of the muscle rehabilitation progress works out better in the relaxed muscular state. In this state, for subject 1, the considered relative difference in muscular frequencies subsequently decreases from 0.297 (in August 2022) to −0.002 (in September 2024). For subject 2, this decrease in the relaxed state is from 0.111 (in January 2024) to 0.038 (in May 2024). However, in February 2024, unexpected results were obtained for subject 2 in both muscular states, which were considered outliers and had been excluded from the analysis. They are, however, shown in Figure 4. For subject 3, this decrease in the relaxed state is from 0.05 (in July 2024) to 0.026 (in November 2024). The improvement in the muscle condition, as observed by natural frequencies analysis, is confirmed by the torque measurement in the knee joints while the extensor muscles are in action. The values of a relative peak torque difference between the reference and ACL legs tend to decrease within the rehabilitation time (see Figure 4).

The results of the muscular tense state also show a tendency to equalization of the RF muscle natural frequency between both legs within rehabilitation progress; however, only for subject 1, the relative difference between the two frequencies subsequently decreases from 0.207 to −0.046 within the observation period. In the cases of subjects 2 and 3, the frequency change indicator increases between subsequent time points. Analysis of the muscular tense state is more complex than that of the relaxed state. The muscle tension was subjective, and moreover, it relates to the current ability of a patient to perform tension, such as fatigue and physical and mental wellbeing, . Attempts were made to standardize the muscular tension by applying a constant load to a lower limb; however, it did not improve the results.

In the relaxed muscular state, *D*_*f*_ reveals a clear decreasing trend over time, corresponding with rehabilitation progress in ACL subjects (Figure 4). Moreover, *D*_*f*_ of ACL subjects approach the *D*_*f*_ of the reference group, which may support the research hypothesis. The significance of mean differences between measurement time points was *p* = 0.138, and in the case of differences between groups, it was *p* = 0.192. This indicates a lack of statistically significant difference between the results obtained at different time points and between the results obtained in both groups of subjects. Although the statistical analysis does not confirm the significance of the differences in the means of *D*_*f*_ within the two factors studied due to the small sample and because the power of the test was low, *p* = 0.232, the obtained mean values of *D*_*f*_ suggest a trend of decreasing values with the progress of rehabilitation.

The observed variation in natural frequencies between both legs proves that, especially at the first time point, the two considered muscles were, in fact, distinct structures physically, which is illustrated by results obtained in the relaxed state, and also functionally, which is illustrated by the results obtained for the tense state. The results presented for the tense state indicate that the RF muscle in the ACL leg could not yield increased tension similar to that observed in the reference leg muscle. This finding aligns with the results of torque in the knee joint measurements and is also consistent with findings of Ithurburn et al. (2018) and Konishi et al. (2012).

The study presents a new method for monitoring the functional recovery of a muscle through contactless and non-invasive measurement of its fundamental natural frequency. There are some existing methods for assessing the functional status of muscles, such as joint torque measurement, myotonometry, EMG, and shear wave elastography. Joint torque measurement is considered the gold standard for assessing muscle strength, offering high precision and exact force data (Fares et al., 2022). However, this method requires specialized, expensive equipment (often exceeding tens of thousands of dollars) and laboratory conditions, which limit its availability in clinical and sport applications. In addition, these tests are time-consuming and require full cooperation from the subject, which may be difficult for patients with injuries or for elderly people. Myotonometry, as a method for assessing muscle tension and elasticity, is characterized by good repeatability and relatively low cost, but its accuracy may be limited when assessing deeper muscles and is susceptible to variable testing conditions (Aird et al., 2012). Shear wave elastography is an innovative technique that allows for precise assessment of muscle stiffness, but due to the high cost of equipment and the need for specialized training, its use in everyday practice is limited (Jonathon et al., 2022). Finally, EMG, both surface and needle, enables the assessment of electrical activity in muscles and is widely used in the study of neuromuscular function. This method allows for the analysis of activation time, motor unit recruitment level, and muscle fatigue (Merletti and Farina, 2016). Although EMG provides valuable physiological data, its interpretation can be complex, and the results are susceptible to cross-talk from neighboring muscles, signal variability, and the requirements of the standard procedure of electrode mounting. In addition, operator experience and appropriate testing conditions are necessary for obtaining reliable measurements.

To evaluate the potential of the presented methodology for assessing muscle functional status, a well-established existing methodology was applied to all subjects, i.e., muscle strength measurement in a lower limb using the Biodex system, which is considered a gold standard in muscle strength assessment. Application of the two methods led to similar conclusions regarding the muscle functional improvement. Thus, the methodology proposed in the present paper shows potential to present similar efficiency in muscular status assessment as presented by the Biodex system. In parallel, the presented approach is certainly cheaper (in terms of cost of equipment), portable, does not demand a dedicated room, and is safer for the patient as research points out that no muscular tension is required to obtain reasonable data and can be conducted soon after the operation. Moreover, the device does not require advanced operator training, which allows for fast and repeatable measurements in various conditions, both in clinics and sports. Due to these benefits, the approach can be integrated into existing rehabilitation protocols more easily than the Biodex device. The new approach has potential for application in rehabilitation progress monitoring, assessing the functional status of muscles in patients after injury, and evaluating muscular readiness to undertake sport challenges, especially in the early stages of post-injury assessment when limb stress is restricted.

The approach, however, has some limitations. Although the paper discusses muscle vibrations, due to measurements of the outer body layer, the collected response signals are, in fact, vibrations of a muscle–fat composite, in which the subcutaneous fat is the outer layer. To minimize its influence on this composite’s response, slim subjects have been selected for the analysis. The percentage of fat in their total body mass has been estimated using bioelectrical impedance analysis. In the future study, vibration characteristics of fat alone should be recognized by vibration measurements conducted for obese subjects. This can help assess the subcutaneous fat influence on the vibration response of a muscle measured on the skin. Second, the symmetry assumption of RF mechanical properties in both legs of a patient may be questionable. Shen et al. (2024) showed an approximate average difference of 0.6 Hz in the natural frequency of RF in the dominant and non-dominant leg in 40 Chinese women with knee osteoarthritis, identified using myotonometry. However, symmetrical response between sides in the quadriceps of healthy older male subjects was also shown using the same device (Aird, Samuel, and Stokes, 2012). These contrary findings indicate that larger studies on the muscles’ free vibrations are warranted to provide reference data for various anthropological groups.

## Data Availability

The raw data supporting the conclusions of this article will be made available by the authors, without undue reservation.
