# Supplementary material for: A novel approach to monitoring rehabilitation progress in atrophic muscle using contactless measurement of free oscillations and advanced modal analysis
Source: Front Bioeng Biotechnol. 2025 Aug 1;13:1496739. doi: 10.3389/fbioe.2025.1496739 (PMC12355181; doi:10.3389/fbioe.2025.1496739)
Supplement: Supplementary file 1 [file DataSheet1.pdf]

Supplementary Material

Exemplary stabilization diagrams in ERA conducted for muscle vibration signals

The Fig. S1 presents stabilization diagram in the ERA procedure applied to signals measured for Subject 1 in third time point for both legs and rectus femoris in two considered states.

It depicts a relatively wide vibrations spectrum, represented here by power spectral density function (PSD) and several frequencies identified by the ERA technique as natural frequencies of a system. They are marked by black crosses, denoting MAC and damping criteria satisfaction. The marks occur several times for each natural frequency, as several orders of eigenvalue problem are considered.

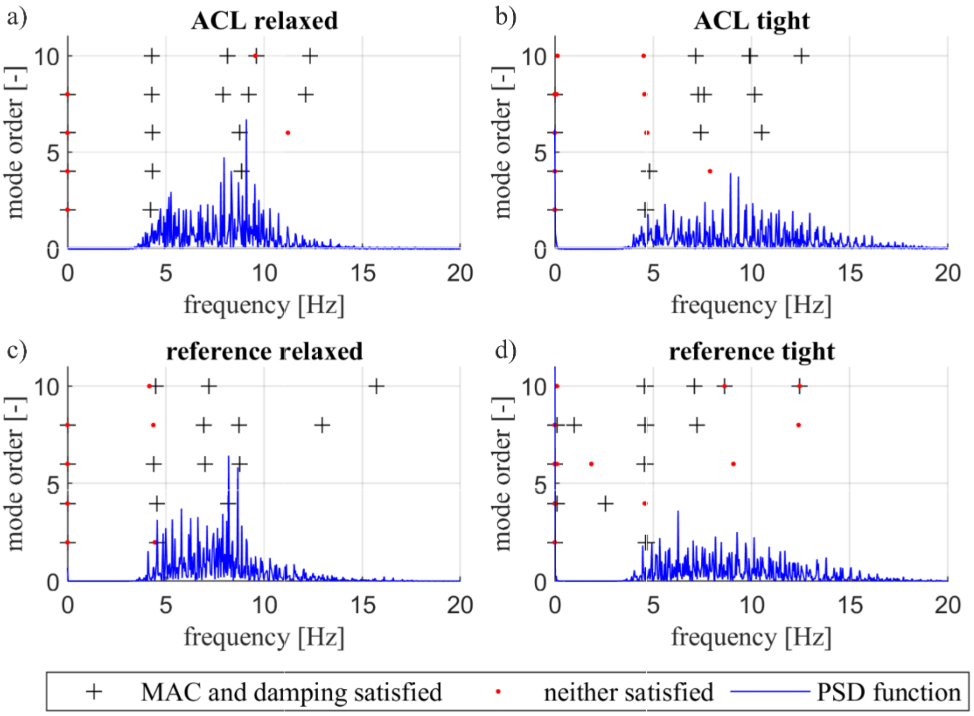

Fig. S1. Stabilization diagram in the ERA procedure applied to signals measured for Subject 1 in the third time point in: (a) ACL leg, RF relaxed; (b) ACL leg, RF tight; (c) reference leg, RF relaxed; (d) reference leg, RF tight

The oscillations frequencies identified in RF muscles in both legs, across all time points and peak torque values for subsequent subjects

For ACL subjects $D_f = \frac{f_r - f_a}{f_r}$, $D_T = \frac{T_r - T_a}{T_r}$;

For reference subjects $D_f = \frac{\left|f_r - f_l\right|}{\max\left(f_r, f_l\right)}$, $D_T = \frac{\left|T_r - T_l\right|}{\max\left(T_r, T_l\right)}$.

Table 1. Natural frequencies of rectus femoris in ACL (f_a) and reference legs (f_r) and peak torque measured for reference (T_r) and ACL (T_a) legs in subsequent time points. Subject 1

Time point	Muscular state	$f_r \pm df_r$ [Hz]	$f_a \pm df_a$ [Hz]	D_f	T_r [Nm]	T_a [Nm]	D_T
VIII.2022		11.92±1.34	8.37±0.47	0.297	-	-	-
IX.2022	relaxed	7.74±0.29	7.06±0.09	0.088	102	72	0.293
IX.2023		8.75±0.74	8.22±0.26	0.053	97	105	-0.082
IX.2024		8.66±1.96	8.67±1.70	-0.002	108	97	0.099
VIII.2022		13.69±3.83	10.85±3.00	0.207			
IX.2022	tense	14.54±0.64	13.06±0.43	0.102			
IX.2023		11.37±1.62	12.08±1.39	-0.062			
IX.2024		9.03±2.04	9.44±2.13	-0.046			

Table 2. Natural frequencies of rectus femoris in ACL (f_a) and reference legs (f_r) and peak torque measured for reference (T_r) and ACL (T_a) legs in subsequent time points. Subject 2

Time point	Muscular state	$f_r \pm df_r$ [Hz]	$f_a \pm df_a$ [Hz]	D_f	T_r [Nm]	T_a [Nm]	D_T
17.I.2024		7.53±1.05	6.69±0.44	0.111	-	-	-
28.II.2024	relaxed	7.82±1.40	8.42±1.34	-0.076	198	103	0.480
10.IV.2024		8.12±1.24	7.35±0.71	0.095	213	179	0.160
22.V.2024		8.60±1.24	8.27±1.38	0.038	202	168	0.168
17.I.2024		10.48±2.81	10.24±2.58	0.022			
28.II.2024	tense	9.78±2.84	7.61±2.13	0.222			
10.IV.2024		8.92±2.51	9.27±2.96	-0.039			
22.V.2024		8.61±2.62	8.31±2.47	0.035			

Table 3. Natural frequencies of rectus femoris in ACL (f_a) and reference legs (f_r) and peak torque measured for reference (T_r) and ACL (T_a) legs in subsequent time points. Subject 3

Time point	Muscular state	$f_r \pm df_r$ [Hz]	$f_a \pm df_a$ [Hz]	D_f	T_r [Nm]	T_a [Nm]	D_T
10.VII.2024	relaxed	8.24±1.94	7.83±0.75	0.050	-	-	-
30.VIII.2024		8.57±1.03	8.29±1.33	0.033	259	127	0.51
19.XI.2024		7.44±1.05	7.24±1.11	0.026	300	215	0.28
10.VII.2024	tense	9.18±2.54	8.42±0.49	0.083			
30.VIII.2024		8.15±1.77	9.08±1.99	-0.114			
19.XI.2024		9.96±2.42	10.50±2.97	-0.054			

Table 4. Natural frequencies of rectus femoris in both legs: right (f_r) and left (f_l) and peak torque measured for right (T_r) and left (T_l) legs in reference subjects.

Subject	Muscular state	$f_r \pm df_r$ [Hz]	$f_l \pm df_l$ [Hz]	D_f	T_r [Nm]	T_l [Nm]	D_T
4	relaxed	7.50±1.30	7.51±1.79	0.0013	249	197	0.209
5		9.04±1.31	9.42±1.10	0.041	148	116	0.216
6		9.18±1.72	9.82±1.72	0.065	193	204	0.054
7		8.16±2.26	8.55±1.92	0.046	197	195	0.010
4	tense	7.36±1.37	6.90±0.83	0.064			
5		9.66±2.49	9.20±3.72	0.047			
6		10.88±4.24	10.57±2.68	0.028			
7		9.60±2.80	10.85±3.32	0.12			
